# Serum alkaline phosphatase relates to cardiovascular risk markers in children with high calcium-phosphorus product

**DOI:** 10.1038/s41598-018-35973-5

**Published:** 2018-12-14

**Authors:** Sílvia Xargay-Torrent, Núria Espuña-Capote, Mercè Montesinos-Costa, Anna Prats-Puig, Gemma Carreras-Badosa, Ferran Díaz-Roldán, Francis De Zegher, Lourdes Ibáñez, Judit Bassols, Abel López-Bermejo

**Affiliations:** 1 Pediatric Endocrinology group, (Girona Biomedical Research Institute) IDIBGI, Girona, Spain; 2 Department of Pediatrics, Dr. Trueta University Hospital, Girona, Spain; 3 Clinical Laboratory, Dr. Trueta University Hospital, Girona, Spain; 4 Department of Physical Therapy, EUSES University School, University of Girona, Girona, Spain; 5 Department of Development & Regeneration, University of Leuven, Leuven, Belgium; 6 Endocrinology Department, Pediatric Research Institute Sant Joan de Déu, University of Barcelona, Barcelona, and Centro de Investigación Biomédica en Red de Diabetes y Enfermedades Metabólicas Asociadas (CIBERDEM), ISCIII, Madrid, Spain; 7 Maternal-Fetal Metabolic group (Girona Biomedical Research Institute), IDIBGI, Girona, Spain

## Abstract

Although alkaline phosphatase (ALP) correlates with cardiovascular risk in adults, there are no studies in children. We evaluated the association between serum ALP levels, calcium-phosphorus product (Ca*P) and cardiovascular risk markers in healthy children. Children aged 7.9 ± 1.4 (n = 379) were recruited in this cross-sectional study. The main outcome measures were systolic and diastolic blood pressure (SBP and DBP) and carotid intima-media thickness (cIMT). Additional assessments were body-mass index (BMI), waist circumference, homeostatic model assessment of insulin resistance (HOMA-IR) and fasting lipids, ALP, serum calcium, phosphorus and Ca*P. ALP was directly correlated with BMI (*p* < 0.0001), waist circumference (*p* < 0.0001), SBP (*p* < 0.0001), cIMT (*p* = 0.005), HOMA-IR (*p* < 0.0001), and fasting triglycerides (*p* = 0.0001). Among them, in children with Ca*P values above the median the associations were BMI (r = 0.231; *p* = 0.001), waist (r = 0.252; *p* < 0.0001), SBP (r = 0.324; *p* < 0.0001), cIMT (r = 0.248; p = 0.001) and HOMA-IR (r = 0.291; *p* < 0.0001)]. ALP independently associated with SBP (β = 0.290, *p* < 0.001) and cIMT (β = 0.179, *p* = 0.013) in children with higher Ca*P, after adjusting for confounding variables. Circulating ALP is associated with a more adverse cardiovascular profile in children with higher Ca*P. We suggest that serum ALP and Ca*P levels could contribute to the assessment of risk for cardiovascular disease in children.

## Introduction

Cardiovascular disorders have become a major public health concern due to their high prevalence in the adult population, thus their prevention during childhood is crucial. Consequently, gaining insight into early processes of disease and discovering new biomarkers for early intervention may turn out to be valuable.

Alkaline phosphatase (ALP) is widely expressed, most abundantly in bone, liver and kidneys. Circulating ALP originates mostly from bone and liver in adults, and predominantly from bone from birth to adolescence^1^. ALP typically catalyses the removal of the phosphate group from diverse phosphate-containing molecules, among other reactions^1^. Physiologically, one of the main roles of ALP is to help in the mineralization of hard tissues, the process whereby hydroxyapatite is deposited in the extracellular matrix, as it supplies the required phosphorus pool^2^.

In adults, positive correlations of ALP with waist circumference, blood pressure and serum triglycerides have been described, although these associations have not been adjusted for BMI^3^. Indeed, obese adults have higher concentrations of circulating ALP^4^. High circulating ALP, however, has been related to cardiovascular and coronary heart disease events independently of body-mass index (BMI), systolic blood pressure (SBP) or serum triglycerides^3,5^. Additional studies in older adults have also supported that high ALP levels associated with an increased risk of 1.19 and 1.10 of coronary heart disease and cardiovascular mortality, respectively, after adjustment for BMI^6^. In adult patients with kidney disease high levels of serum ALP were also linked to increased cardiovascular-related hospitalization^7^. No studies, however, have been reported in children.

Both calcium and phosphorus are essential substrates involved in the mineralization process. Whereas physiological calcification occurs in hard tissues, the same process can occur pathologically in soft tissues. Serum calcium-phosphorus product (Ca*P) has been linked to vascular calcification, and thus been regarded as a risk factor for extra-skeletal calcification^8^. For example, an elevated serum Ca*P concentration is considered to be a risk factor for coronary artery disease in adults with metabolic syndrome^9^.

In this context, our aim is to study whether circulating ALP concentrations are related to cardiovascular risk markers in school-aged children, particularly in those with higher Ca*P. As a secondary aim, we study the associations of ALP with anthropometric and metabolic parameters as they might act as confounding factors in cardiovascular risk assessment.

## Results

Results for anthropometric, metabolic and cardiovascular parameters are shown in Table 1 for all the studied children (n = 379) enrolled in the study, and for subgroups thereof according to the median of Ca*P. None of the variables significantly differed among low and high Ca*P subgroups, except for age, HDL cholesterol and, as expected, calcium and phosphorus levels (Table 1).

**Table 1:** Anthropometric, metabolic and cardiovascular variables in the studied subjects and in subgroups according to the median of the calcium and phosphorus product (Ca*P).

	All subjects	Ca*P below the median	Ca*P above the median
n	379	189	190
Gender (female, %)	49.6	47.6	51.6
Age (years)	7.9 ± 1.4	8.1 ± 1.4	7.6 ± 1.5^b^
Puberty (Tanner >1, %)	13.2	13.2	13.2
BMI (kg/m2)	19.5 ± 4.2	19.6 ± 4.4	19.3 ± 4.0
BMI-SDS (z-score)	0.67 ± 1.45	0.65 ± 1.46	0.70 ± 1.44
Waist (cm)	65 ± 12	65 ± 12	64 ± 12
Fat mass (%)	30.2 ± 9.9	30.7 ± 10.2	29.7 ± 9.4
SBP (mmHg)	106 ± 10	106 ± 11	105 ± 10
DBP (mmHg)	61 ± 8	61 ± 8	61 ± 7
cIMT (mm)	0.041 ± 0.007	0.041 ± 0.007	0.040 ± 0.007
Insulin (mcU/ml)	5.2 ± 4.7	5.4 ± 5.0	5.0 ± 4.4
HOMA-IR	1.1 ± 1.1	1.2 ± 1.2	1.1 ± 1.0
HDL cholesterol (mg/dL)	58 ± 15	56 ± 14	60 ± 15^a^
Triglycerides (mg/dL)	61 ± 31	63 ± 33	60 ± 29
Alkaline phosphatase (U/L)	240 ± 55	241 ± 58	240 ± 52
Calcium (mg/dL)	9.9 ± 0.3	9.8 ± 0.3	10.0 ± 0.3^b^
Phosphorus (mg/dL)	4.9 ± 0.4	4.6 ± 0.3	5.2 ± 0.3^b^
Ca*P	49 ± 5	45 ± 3	52 ± 3^b^

Data are shown as mean ± SD for quantitative variables.

BMI, body mass index; SBP, systolic blood pressure; DBP, diastolic blood pressure; cIMT, carotid intima-media thickness; HOMA-IR, Homeostatic model assessment of insulin resistance; HDL, high-density lipoprotein; Ca*P, calcium-phosphorus product.

^a^*p* < 0.05; ^b^*p* < 0.001 for differences by Student’s t-test.

As regards the whole sample of children, serum ALP levels were related to various metabolic and cardiovascular parameters. Specifically, in the whole sample of subjects, ALP showed positive correlations with BMI, BMI-SDS, waist circumference, SBP, cIMT, insulin, HOMA-IR and fasting triglycerides (Table 2); and to a lesser extent, associations were also found with age, fat fraction and DBP (Table 2).

**Table 2:** Pearson correlation coefficients for alkaline phosphatase and selected variables in the studied subjects and in subgroups according to the median of calcium and phosphorus product (Ca*P).

	All subjects (n = 379)		Ca*P below the median (n = 189)		Ca*P above the median (n = 190)	
	r	*p-*value	r	*p-*value	r	*p-*value
Age (years)	0.104	0.042	0.010	0.893	0.205	0.005
BMI (kg/m^2^)	0.186	<0.001	0.148	0.042	0.231	0.001
BMI-SDS (z-score)	0.176	0.001	0.150	0.039	0.205	0.005
Waist (cm)	0.202	<0.001	0.159	0.028	0.252	<0.001
Fat mass (%)	0.136	0.008	0.123	0.093	0.151	0.037
SBP (mmHg)	0.214	<0.001	0.125	0.086	0.324	<0.001
DBP (mmHg)	0.110	0.032	0.103	0.158	0.119	0.102
cIMT (mm)	0.145	0.005	0.053	0.469	0.248	0.001
Insulin (mcU/ml)	0.210	<0.001	0.156	0.032	0.280	<0.001
HOMA-IR	0.211	<0.001	0.150	0.040	0.291	<0.001
HDL cholesterol (mg/dL)	−0.062	0.226	−0.073	0.318	−0.051	0.485
Triglycerides (mg/dL)	0.178	0.001	0.215	0.003	0.129	0.075
Calcium (mg/dL)	0.094	0.067	0.084	0.251	0.125	0.087
Phosphorus (mg/dL)	−0.036	0.489	−0.004	0.962	−0.098	0.181
Ca*P	0.000	0.993	0.045	0.535	−0.030	0.679

BMI, body mass index; SBP, systolic blood pressure; DBP, diastolic blood pressure; cIMT, carotid intima-media thickness; HOMA-IR, Homeostatic model assessment of insulin resistance; HDL, high-density lipoprotein; Ca*P, calcium-phosphorus product.

**Figure 1:**
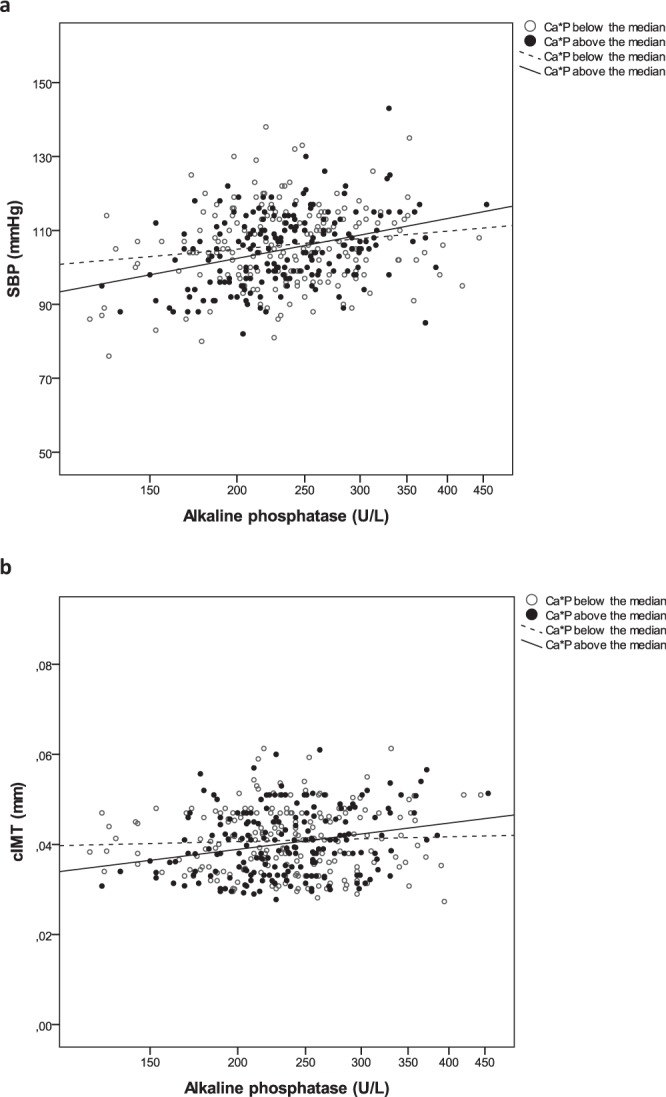
Correlation graphs between alkaline phosphatase (ALP) and cardiovascular risk markers in children. (**A**) Relationship between serum ALP and systolic blood pressure (SBP) in subgroups thereof according to the Ca*P median (Below the median r = 0.125, p = 0.086; above the median r = 0.324 p < 0.0001). (**B**) Relationship between serum ALP and carotid intima-media thickness (cIMT) in subgroups thereof according to the median of the calcium and phosphorus product (Ca*P; below the median r = 0.053, p = 0.469; above the median r = 0.248, p = 0.001).

When dividing the sample of children by Ca*P groups, the ALP associations with BMI, BMI-SDS, waist circumference, insulin, and HOMA-IR were independent of the Ca*P group (Table 2). Serum triglycerides positively correlated with ALP only in the lower Ca*P group (r = 0.215; p = 0.003). Within the higher Ca*P group, circulating ALP levels were specifically associated with age (r = 0.205; p = 0.005), fat mass (r = 0.151; p = 0.037), SBP (r = 0.324; *p* < 0.0001; Fig. 1A) and cIMT (r = 0.248; *p* = 0.001: Fig. 1B; Table 2).

Next, we investigated whether ALP was preferentially related to SBP and cIMT in children with higher Ca*P rather than in the lower Ca*P group. The interaction between ALP and Ca*P was significant after ANCOVA test for cIMT (*p* = 0.037, Table 3), confirming that the correlation between ALP and cIMT was stronger in children with higher Ca*P (r = 0.248) than in those with lower Ca*P (r = 0.053; Table 2). For SBP, the ANCOVA test did not reach statistical significance (p = 0.050, Table 3).

**Table 3:** ANCOVA analysis testing the interaction of calcium and phosphorus product (Ca*P) in the association of alkaline phosphatase and SBP/cIMT (n = 379).

	SBP		cIMT	
	F	p-value	F	p-value
Ca*P	4.62	0.032	4.93	0.027
Alkaline phosphatase	19.79	<0.001	9.44	0.002
Ca*P x Alkaline phosphatase	3.87	0.050	4.37	0.037

SBP, systolic blood pressure; cIMT, carotid intima-media thickness; Ca*P, calcium-phosphorus product.

Finally, in multivariable regression analyses adjusting for confounding variables (age, puberty, gender, BMI, HOMA-IR and triglycerides), ALP was shown to be independently associated with SBP (β = 0.290; *p* < 0.0001, R^2^ = 0.10), and with cIMT (β = 0.179; *p* < 0.013, R^2^ = 0.04; Table 4) in high Ca*P children.

**Table 4:** Multivariable regression analysis of SBP and cIMT as dependent variables in the studied subjects and in subgroups thereof according to the median of calcium and phosphorus product (Ca*P).

	β	*p*-value	R^2^
**SBP as dependent variable**			
All children (n = 379)		<0.001	0.14
BMI	0.317	<0.001	0.12
Alkaline phosphatase	0.156	0.002	0.02
Ca*P below the median (n = 189)		<0.001	0.21
BMI	0.396	<0.001	0.20
Triglycerides	0.142	0.046	0.01
Ca*P above the median (n = 190)		<0.001	0.12
Alkaline phosphatase	0.295	<0.001	0.10
Age	0.156	0.028	0.02
**cIMT as dependent variable**			
All children (n = 379)		<0.001	0.06
BMI	0.217	<0.001	0.05
Alkaline phosphatase	0.110	0.032	0.01
Ca*P below the median (n = 189)		<0.001	0.08
BMI	0.228	0.001	0.05
Female gender	0.199	0.005	0.03
Ca*P above the median (n = 190)		<0.001	0.11
Age	0.166	0.026	0.06
Alkaline phosphatase	0.180	0.014	0.04
BMI	0.150	0.046	0.01

Variables included in the model were the following: age, puberty, gender, BMI, SBP, HOMA-IR, triglycerides and alkaline phosphatase.

## Discussion

Circulating ALP was found to relate to cardiovascular risk markers, such as SBP and cIMT, in school-aged children with higher circulating Ca*P. Serum ALP and Ca*P concentrations might thus contribute in the assessment of cardiovascular risk during childhood.

The present study appears to be the first to have related serum ALP levels to cardiovascular risk factors in children. Supporting our data, results reported in adults, showed that high ALP levels correlated with the risk of developing cardiovascular disease^3,6^ and total mortality^5,10^. Additionally, SBP and cIMT were also shown to be positively associated with serum ALP levels in hypertensive men^11^.

In bone, maturation of osteoblasts is coupled to the release of ALP-containing microvesicles that promote mineral deposition. Equivalent structures have been found in vascular tissue^12^. Metabolic imbalances, such as elevated calcium or phosphate levels, stimulate the up-regulation of several osteogenic markers in vascular smooth muscle cells, together with the secretion of the calcifying microvesicles^13–16^. This effect, coupled to downregulation of mineralization inhibitors in serum, eventually leads to vascular calcification^15^. Interestingly, ALP hydrolyses a major mineralization inhibitor, the inorganic pyrophosphate, thus regulating the propagation of mineralization^17–20^. Under pathological conditions, the enzyme contributes to the specific calcification of the medial layer of vasculature^21^, which triggers vascular changes as assessed by cIMT^22^. In a context of mineral homeostasis dysregulation in children, such as in renal diseases, higher serum ALP levels have been linked consistently to increased cIMT^23,24^. Together, these data fit with the present finding that higher ALP concentrations relate to greater cIMT in children with higher Ca*P.

Medial vascular calcification is similar to bone mineralization^25^ and causes concentric calcification of the blood vessel and elastinolysis^26^. This process has substantial cardiovascular consequences, resulting in vascular stiffening, reduced compliance and elastance, and ultimately increasing SBP and cardiac workload, and causing left ventricular cardiac hypertrophy and heart failure^26–31^. Thus, it seems plausible that due to early vascular calcification, SBP may be elevated. Accordingly, serum ALP levels have been associated with markers of vascular function and blood pressure in adults with and without hypertension^11,32^.

Ca*P levels seem to be influenced by age in our sample. Children between 7 and 10 years old may be using high levels of calcium for bone growth^33^, however calcium levels are rather stable during childhood^34^. On the contrary, phosphorus levels decrease when infants and children grow old^34,35^, thus affecting Ca*P levels. Therefore, our subsequent results have been adjusted for age to correct for this physiological effect. Our results did not reveal Ca*P as a cardiovascular risk factor since both groups of children showed a similar cardiovascular profile. Moreover, the role of Ca*P as a risk factor for cardiovascular diseases has been controversial^36^. Instead, the interaction between the Ca*P and ALP levels would indicate the outcome, thus in the high Ca*P group, ALP levels associated positively with cardiovascular risk markers (SBP and cIMT) but not in low Ca*P subgroup. This suggests the requirement of both elevated Ca*P and elevated ALP to render a poorer cardiovascular profile. As a consequence, when ALP or Ca*P were raised alone, there were no effects on SBP or cIMT. It is thus possible that high Ca*P primes children to develop vascular calcification while high ALP levels trigger the process^12^.

Study limitations include: (i) bone and liver isoforms of ALP were not discriminated, although, as said, most ALP derives from bone in children, (ii) the cross-sectional study design, which excluded proof of causality in the relationship between circulating ALP and either SBP or cIMT, (iii) relatively weak correlations of these clinical associations; however, taken into account that this is a population of healthy children, we believe they are notable and expect to find stronger correlations in children at higher risk for cardiovascular disease, such as those with a positive family history for this disease.

Whether the associations suggested by our results could be applied to the clinic, as part of a set of biomarkers to assess cardiovascular risk at childhood, should be confirmed in appropriate longitudinal studies. Moreover, the limitations of using ALP as a biomarker have to be explored as well, since growth spurts/puberty in children may result in highly variable ALP.

In summary, higher serum ALP levels were found to associate with a more unfavourable cardiovascular profile in children with higher Ca*P. Further studies are warranted to confirm whether circulating ALP and Ca*P levels might help in the detection of early vascular damage, and contribute to the development of paediatric strategies aimed at preventing cardiovascular disease in adulthood.

## Methods

### Population and ethics

A sample of school-aged children (n = 379), without a family history of cardiovascular disease (as assessed by interviewing the parents), participated in the study. Subjects were consecutively recruited among those seen in a primary care setting in Northeastern Spain [(mean age of 7.9 ± 1.4 years; mean body mass index (BMI)-standard deviation score (SDS) of 0.67 ± 1.45]. Puberty was assessed by a specifically trained nurse using Tanner criteria. Prepubertal children were those in Tanner stage I. Exclusion criteria were: major congenital anomalies; abnormal blood count; abnormal liver, kidney or thyroid functions; chronic illness or prolonged use of medication; acute illness or use of medication during the month previous to the potential enrolment. The study protocol was approved by the Institutional Review Board of Dr Josep Trueta Hospital and was carried out according with The Code of Ethics of the World Medical Association (Declaration of Helsinki). Informed written consent was obtained from the parents. All data generated or analysed during this study are included in this published article.

### Clinical assessments

Clinical examination was carried out in the morning. A calibrated scale and a Harpenden stadiometer were used to obtain weight and height measures, respectively. BMI was calculated with the following formula: weight in kg/(height in meters)^2^. BMI-SDS adjusted for age and sex, was computed using regional normative data^37^. Waist circumference at the umbilical level was measured in the supine position. Body composition was determined by bioelectric impedance (Hydra Bioimpedance Analyzer 4200, Xitron Technologies) and fat mass percentage was quantified with the formula: Fat mass = (body weight − lean mass)/*100. After a 10-minute rest, blood pressure was taken on the right arm with the child supine, using an electronic sphygmomanometer (Dinamap Pro 100, GE Healthcare).

### High-resolution ultrasound measurement of carotid intima-media thickness

Carotid intima-media thickness (cIMT) was assessed by high-resolution ultrasonography (MyLabTM25, Esaote). Images were obtained using a linear 12-MHz transducer on the right side at the level of the distal common carotid artery, one centimetre away from its bifurcation. The cIMT value was computed as the average of 5 measurements. Intra-subject coefficient of variation was below 6%. None of the children included in the study exhibited visible signs of calcification as assessed by ultrasound.

### Laboratory variables

Blood sampling was carried out in the morning under fasting conditions. Serum glucose was quantified by the hexokinase method. Insulin was detected by immunochemiluminiscence (Immulite 2000, Diagnostic Products). The limit of detection was 0.4 mIU/L and coefficient of variation (CV) was less than 10%. Homeostatic model assessment of insulin resistance (HOMA-IR) index was calculated according to the formula: fasting insulin (µU/mL) × fasting glucose (mg/dL)/405. Total triglycerides (TG) were quantified with glycerolphosphate oxidase (ARCHITECT, Abbott Laboratories), with detection threshold of 5 mg/dL and CV below 5%. A homogeneous method of selective detergent with accelerator (ARCHITECT, Abbott Laboratories) was used to determine HDL-cholesterol levels, with detection limit and CV of 2.5 mg/dL and inferior to 4%, respectively. ALP was quantified by colorimetrical detection of p-nitrophenyl phosphate product, with a limit of detection of 5 U/L. Inorganic phosphorus was colorimetrically assayed after reacting with ammonium molybdate in acidic medium (detection limit 0.3 mg/dL). Calcium was assessed using the chromophore 5-nitro-5′-methyl-1,2-bis(o-aminophenoxy)ethan-N,N,N’,N’-tetraacetic acid. Ca*P was calculated as the product between serum calcium and phosphorus.

### Statistics

Statistical analyses were performed using SPSS version 22.0 (SPSS Inc.). The study has an 80% power to detect a significant Pearson correlation coefficient of at least 0.15 between ALP levels and cardio-metabolic parameters, accepting an alpha risk of 0.05 in a bilateral contrast (GRANMO, IMIM, version 7.12). Results are expressed as mean ± standard deviation (SD). Median Ca*P value was used to categorize subjects in subgroups with serum Ca*P above versus below the median. Non-normally distributed variables (Kolmogorov-Smirnov normality test) were mathematically transformed to improve symmetry with logarithmic and quadratic functions. Differences between Ca*P subgroups were assessed by Student t-test (continuous data) and by Chi square (categorical data). The relation between variables was tested by Pearson bivariate correlations followed by multivariable linear regression analyses. The stepwise method was used for computing the independent variables. Ca*P interaction in the association between ALP and systolic blood pressure (SBP)/cIMT was assessed by analysis of covariance (ANCOVA). Significance level was set at *p* < 0.05.
